# Opposite effects of HDAC5 and p300 on MRTF-A-related neuronal apoptosis during ischemia/reperfusion injury in rats

**DOI:** 10.1038/cddis.2017.16

**Published:** 2017-02-23

**Authors:** Na Li, Qiong Yuan, Xiao-Lu Cao, Ying Zhang, Zhen-Li Min, Shi-Qiang Xu, Zhi-Jun Yu, Jing Cheng, Chunxiang Zhang, Xia-Min Hu

**Affiliations:** 1 Department of Pharmacy, College of Medicine, Wuhan University of Science and Technology, Wuhan, Hubei Province 430065, China; 2 Drug Research Base of Cardiovascular and Cerebral Vascular, College of Medicine, Wuhan University of Science and Technology, Wuhan, Hubei Province 430065, China; 3 Department of Biomedical Engineering, School of Medicine and School of Engineering, The University of Alabama at Birmingham, Birmingham, AL 35294, USA

## Abstract

Our recent study has revealed that the myocardin-related transcription factor-A (MRTF-A) is involved in the apoptosis of cortical neurons induced by ischemia/reperfusion (I/R). Histone deacetylase 5 (HDAC5) and histone acetyltransferase p300 (P300) are two well-known regulators for transcription factors; however, their roles in MRTF-A-related effect on neuronal injuries during I/R are still unclear. In this study, in a model rat cerebral I/R injury via middle cerebral artery occlusion and reperfusion, we found that the expression and activity of HDAC5 was upregulated, whereas p300 and MRTF-A were downregulated both in expression and activity during I/R. Their expression changes and the interaction of the MRTF-A with HDAC5 or p300 were further verified by double immunofluorescence and co-immunoprecipitation. In cultured neuronal apoptosis model induced by H_2_O_2_, MRTF-A exhibited an anti-apoptotic effect by enhancing the transcription of Bcl-2 and Mcl-1 via CArG box binding. MRTF-A-induced anti-apoptotic effect was effectively inhibited by HDAC5, but was significantly enhanced by p300. The results suggest that both HDAC5 and p300 are involved in MRTF-A-mediated effect on neuronal apoptosis during ischemia/reperfusion injury, but with opposite effects.

Cerebral ischemia is a serious condition associated with vascular disease, affecting patients worldwide. Despite mitigating initial tissue hypoxia, the subsequent restoration of blood flow and reoxygenation is frequently associated with an exacerbation of cerebral tissue injury.^1^ The pathogenesis is complex and involves a myriad of distinct cellular events and multiple molecular pathways. Although the apoptosis is a prominent cellular injury mechanism, understanding the mechanisms underlying cerebral neuron apoptosis is still the key prerequisite for the treatment of brain ischemia/reperfusion (I/R) injuries effectively.^2, 3^

The myocardin-related transcription factors (MRTF) are coactivators of serum-response factor (SRF)-mediated gene expression.^4^ Activation of MRTF-A occurs in response to alterations in gene expression.^5, 6^ MRTF-A forms a ternary complex with the serum-response factor (SRF) bound to the DNA consensus sequence CC(A/T)_6_GG, known as a CArG box.^7^ In our recent study, we have identified for the first time that hydrogen peroxide (H_2_O_2_) downregulates MRTF-A expression and induces apoptosis in cerebral cortex neurons.^8^ This effect depends on the transcriptional effects of MRTF-A on Bcl-2 and Mcl-1 genes. However, how the activity and the expression of MRTF-A is regulated after brain impairment due to I/R is still unknown.

Histone modification and chromatin remodeling have taken the center stage with respect to orchestrating almost every aspect of nuclear transcription factor function in cell proliferation,^9^ apoptosis,^10, 11, 12^ migration, neurogenesis,^13, 14^ and neural network integration.^15, 16^ Histone deacetylases (HDACs) are implicated in chromatin remodeling and subsequent transcription regulation by controlling the status of histone deacetylation, whereas histone acetyltransferases (HATs) determine the post-translational acetylation status of chromatin and a number of other non-histone proteins.^17, 18, 19, 20^ HDAC5, a class II HDAC, has been shown to have a critical role in cell proliferation^21, 22^ and apoptosis^23, 24^ in different tissues. In addition to its major location in nuclear area, HDAC5 could also be exported into cytoplasm in apoptotic neuronal cells treated with N-methyl-D-aspartic acid (NMDA).^25^ Recent studies have shown that the transcriptional activity of myocardin could be positively and negatively modulated by p300, a member of the HATs.^26, 27^ In addition, p300 interacts with myocardin at its C-terminal transactivation domain to enhance the transactivity of myocardin in activating cardiac and smooth muscle gene expression.^28^

Based on these previous reports from us and other groups, we hypothesize that MRTF-A is a key regulator in the neuronal apoptosis during ischemia/reperfusion, and HDAC5 and p300 may achieve their effects on ischemia/reperfusion by a novel molecular mechanism via regulating the activity/expression of MRTF-A.

## Results

### Apoptosis induced by cerebral ischemia/reperfusion (I/R) model

The I/R model was successfully induced and confirmed by TTC stain (Supplementary Figure S1). Apoptosis was detected by TUNEL and caspase-3 cleavage. As shown in Supplementary Figure S2, the TUNEL-positive cells significantly increased in I/R rats (61.8±7.4%) compared with the sham group (5.8±1.3%) (*P*<0.01). Meanwhile, caspase-3 (Supplementary Figure S2c) cleavage was upregulated in the brain of I/R model rats.

### The expression and activity of HDAC5 and p300 in cerebral ischemia/reperfusion model

**Figure 1:**
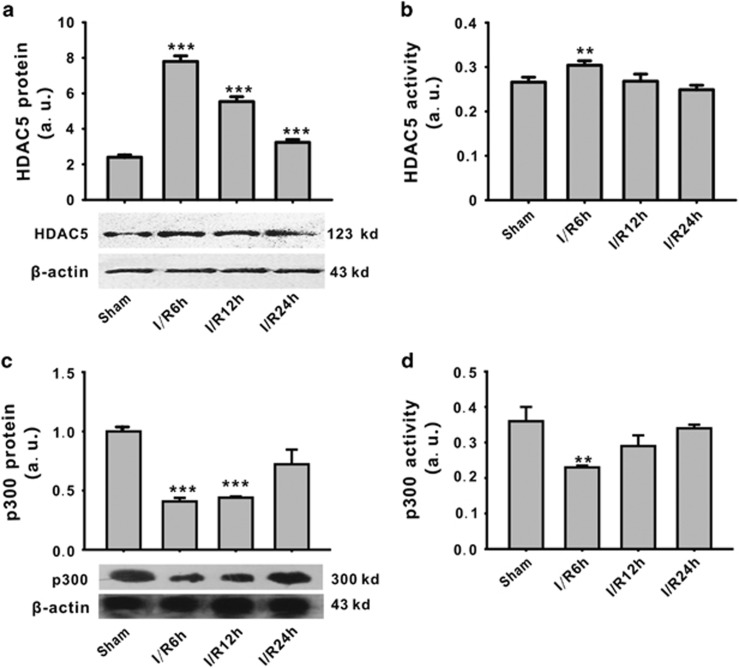
Expression and activity of HDAC5 and p300 protein in ischemia/reperfusion model. HDAC5 protein expression was upregulated (**a**) and its activity was increased (**b**). P300 protein expression was downregulated (**c**) and its activity was inhibited (**d**). **P<0.01, ***P<0.001 *versus* sham. *N*=6 in each group

As a member of histone deacetylase family or histone acetyltransferase, the expression and activity of HDAC5 and p300 were both determined at 6 h, 12 h and 24 h after reperfusion. Compared with the sham group, the expression and activity of HDAC5 were significantly increased at reperfusion 6 h (7.80±0.31 and 0.304±0.10, respectively) and recovered to the normal level at 24 h after reperfusion compared with the sham group (Figures 1a and b). In contrast, the expression and activity of p300 were both significantly decreased at reperfusion 6 h and recovered to normal levels at 24 h after reperfusion compared with the sham group (Figures 1c and d).

### The involvement of MRTF-A/Bcl-2/Mcl-1 downregulated in cerebral ischemia/reperfusion model

**Figure 2:**
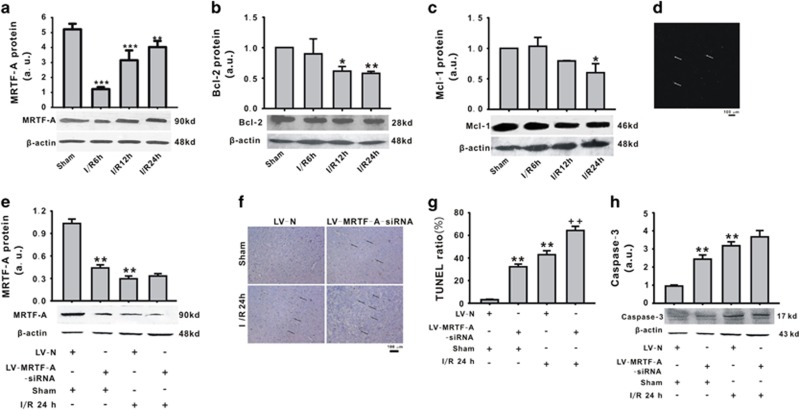
The involvement of MRTF-A/Bcl-2/Mcl-1 downregulated in cerebral ischemia/reperfusion model. MRTF-A (**a**), Bcl-2 (**b**), and Mcl-1 (**c**) protein expression was downregulated. LV-MRTF-A-siRNA was transfected in the brain tissue (**d**, 100 ×). MRTF-A protein expression was detected (**e**). The apoptosis of brain neurons was detected by TUNEL (**f**–**g**, 400 ×) and cleaved caspased-3 (**h**). *P<0.05, **P<0.01, ***P<0.001 *versus* sham; ^++^*P*<0.001 *versus* LV-N+I/R 24 h. *n*=5–6 in each group. LV-N: lentivirus-negative-siRNA; LV-MRTF-A-siRNA: lentivirus-negative-MRTF-A-siRNA

To determine the relationship between MRTF-A and apoptosis in the brain caused by I/R, the expression and activity of MRTF-A were measured. As shown in Figure 2a, MRTF-A protein expression was significantly downregulated after reperfusion which was bottomed at 6 h after reperfusion (*P*<0.01). Interestingly, as the target transcriptional genes of MRTF-A, the expression of Bcl-2 and Mcl-1 was significantly decreased at 24 h after reperfusion compared with the sham group (Figures 2b and c). Maybe, there was a time window between MRTF-A expression and the expression of their target genes, Bcl-2 and Mcl-1.

To further explore the role of MRTF-A in the neuron apoptosis *in vivo*, the expression of MRTF-A was inhibited by LV-MRTF-A-siRNA via ventricle injection (Figure 2d). As shown in Figure 2e, LV-MRTF-A in brain was successfully downregulated by LV-MRTF-A-siRNA in sham group, although no further downregulation was found in I/R group. LV-MRTF-A-siRNA increased the ratio of the apoptosis of brain neurons (32.2±2.4%) in sham group, which was similar with I/R 24 h group (42.9±3.5%, Figure 2g). Compared with the ratio of TUNEL-positive cells (42.9±3.4%) of brain cortex neurons, LV-MRTF-A-siRNA significantly increased the ratio of TUNEL-positive cells (64.3±3.7%, Figure 2g). Additionally, LV-MRTF-A-siRNA increased the effect of I/R 24 h on cleaved caspase-3 level without significance (Figure 2h).

### Relationship of HDAC5 or p300 and MRTF-A induced by ischemia/reperfusion

**Figure 3:**
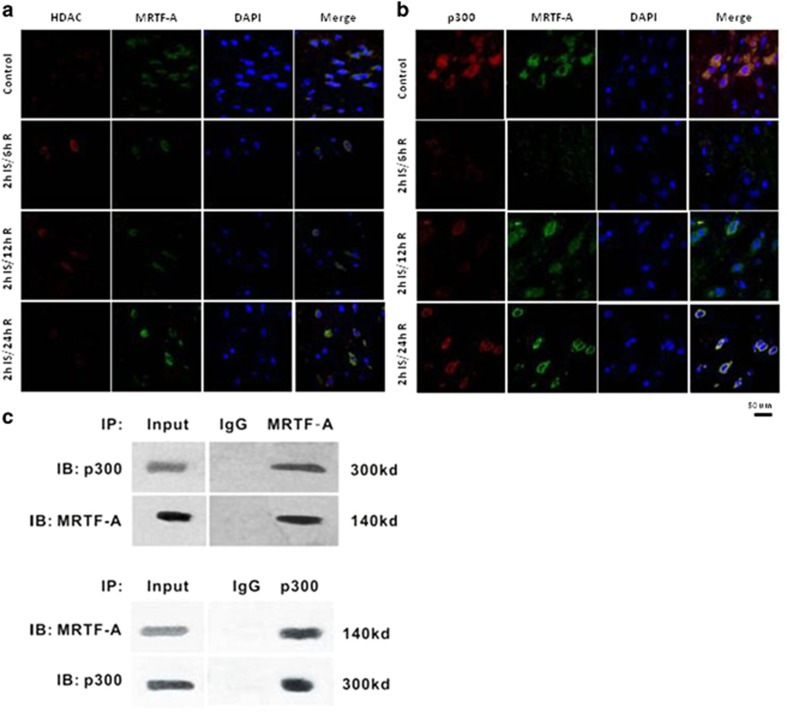
Expression of MRTF-A, HDAC5, and p300 and their localization in rat brain sections. (**a**) Representative images showing the expression and localization of MRTF-A and HDAC5 in sections from control rats and from rats after I/R (400 ×). (**b**) Representative images showing the expression and localization of MRTF-A and p300 in sections from control rats and from rats after I/R. (**c**) The interaction of MRTF-A and p300 by co-immunoprecipitation. Representative images were from six rats of each group. *n*=3. Data are representative of three independent experiments

To explore the relationship of HDAC5 or p300 and MRTF-A, we determined the colocalization of these protein expression by double immunostaining. As shown in Figure 3a, HDAC5 was upregulated, while MRTF-A protein was downregulated during I/R, and these responses recovered at 24 h after reperfusion. P300 and MRTF-A were colocalized both in the nuclear and in cytoplasm (Figure 3b). Both of them were downregulated after ischemia/reperfusion 6 h and recovered after 24 h of reperfusion. The direct interaction of p300 with MRTF-A was further verified by co-immunoprecipitation (IP) as shown in Figure 3c.

### The effects of HDAC5 and p300 on MRTF-A-induced anti-apoptotic effect on neurons

**Figure 4:**
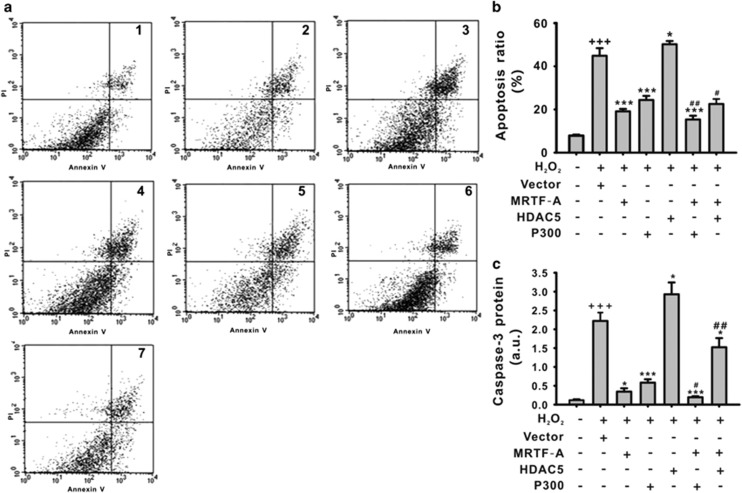
The effect of MRTF-A, HDAC5, and p300 on apoptosis of cortical neurons induced by H_2_O_2_. Apoptosis was detected by Annexin-V+PI double staining (**a** and **b**) and caspase-3 expression (**c**). (1) control, (2) Vector+H_2_O_2_ (400 *μ*M, 24 h), (3) H_2_O_2_ (400 *μ*M, 24 h)+MRTF-A, (4) H_2_O_2_ (400 *μ*M, 24 h) +p300, (5) H_2_O_2_ (400 *μ*M, 24 h)+HDAC5, (6) H_2_O_2_ (400 *μ*M, 24 h)+MRTF-A+p300, (7) H_2_O_2_ (400 *μ*M, 24 h)+MRTF-A+HDAC5. ^+++^*P*<0.001 *versus* control; *P<0.05, ***P<0.001 *versus* H_2_O_2_+vector; ^#^*P*<0.05, ^##^*P*<0.01 *versus* H_2_O_2_+MRTF-A. *n*=4. Data are representative of four independent experiments

To explore the effects of HDAC5 or p300 on MRTF-A-mediated effect on neurons, HDAC5, p300, or MRTF-A was overexpressed in neurons (Supplementary Figure S3). H_2_O_2_ (400 *μ*mol/l, 24 h) induced the apoptosis and caspase-3 expression in cortical neurons (Figures 4a and b). Overexpression of MRTF-A or p300 inhibited the apoptosis of cortical neurons induced by H_2_O_2_. In addition, overexpression of p300 enhanced the MRTF-A-induced anti-apoptotic effect as shown by the apoptotic cell ratio and the caspase-3 expression (Figures 4a–c). In contrast, overexpression of HDAC5 inhibited the anti-apoptotic effect of MRTF-A on the cells (Figures 4a–c).

### The effects of HDAC5 and p300 on MRTF-A-induced expression of its downstream target genes, Bcl-2 and Mcl-1

**Figure 5:**
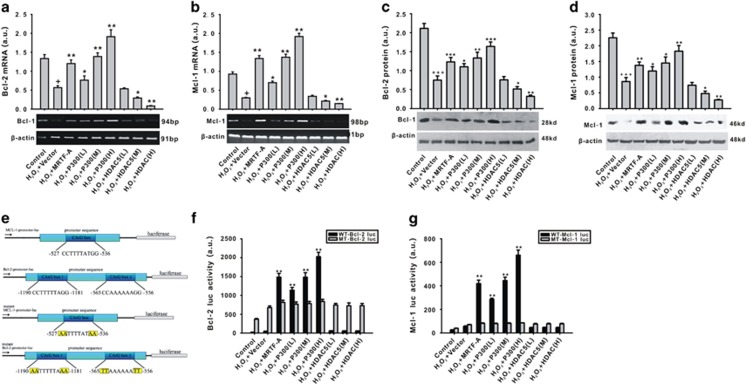
The effects of MRTF-A, HDAC5, and p300 on the expression and transcription of Bcl-2 and Mcl-1 is impaired by H_2_O_2_. The mRNA expression of Bcl-2 (**a**) and Mcl-1 (**b**) were detected using RT-PCR. Protein levels of Bcl-2 (**c**) and Mcl-1 (**d**) were detected by RT-PCR. The wild type and mutant type of CArG box of Bcl-2 and Mcl-1 were established (**e**). The activity of CArG box of Bcl-2 (**f**) and Mcl-1 (**g**) were measured using a luciferase reporter assay. L: the plasmid was transfected at 0.1 *μ*g/8^10^ cell, M: the plasmid was transfected at 0.25 *μ*g/8^10^ cell, H: the plasmid was transfected at 0.5 *μ*g/8^10^ cell, WT: wild type, MT: mutant type. ^+^*P*<0.05 *versus* control; *P<0.05, **P<0.01 *versus* H_2_O_2_+vector. *n*=4. Data are representative of four independent experiments

The anti-apoptotic proteins Bcl-2 and Mcl-1 are two target genes of MRTF-A. As shown in Figure 5, H_2_O_2_ inhibited Bcl-2 and Mcl-1 mRNA and protein expression (Figures 5a–d). Overexpression of MRTF-A or p300 upregulated the expression of Bcl-2 and Mcl-1, whereas the expression of both Bcl-2 and Mcl-1 was effectively inhibited by HDAC5 overexpression (Figures 5a–d). To explore the potential regulatory effect of MRTF-A, p300, and HDAC5 on Bcl-2 and Mcl-1 mRNA transcription, wild-type and mutant promoters were constructed (Figure 5e). We found that H_2_O_2_ inhibited the CArG box transcription activity of the Bcl-2 and Mcl-1 (Figures 5e and f). Interestingly, H_2_O_2_-induced effect on the CArG box transcription activity could be effectively inhibited by the overexpression of MRTF-A or p300, whereas HDAC5 did not have this function (Figures 5e and f). Mutation of the CArG box gene of Bcl-2 and Mcl-1 abolished the effects of MRTF-A and p300 on the CArG box transcription activity.

**Figure 6:**
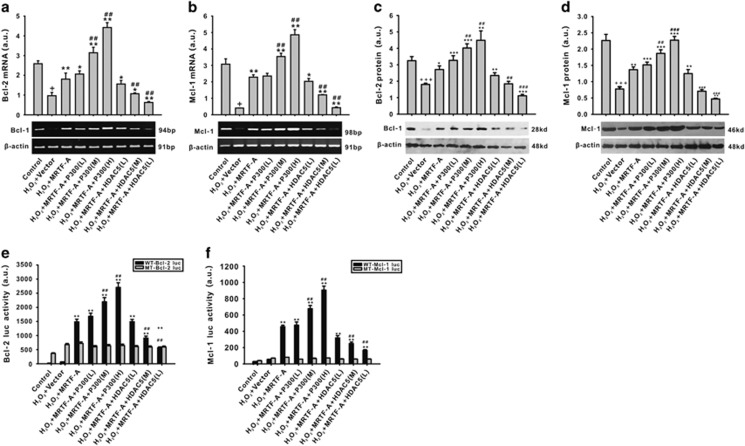
The regulative effects of HDAC5 and p300 on the transcription activity of MRTF-A on Bcl-2 and Mcl-1 expression is impaired by H_2_O_2_. The mRNA expression of Bcl-2 (**a**) and Mcl-1 (**b**) were detected by RT-PCR. Protein levels of Bcl-2 (**c**) and Mcl-1 (**d**) were detected by western blotting. The activity of CArG box of Bcl-2 (**e**) and Mcl-1 (**f**) were measured using a luciferase reporter assay. L: the plasmid was transfected at 0.1 *μ*g/8^10^ cell, M: the plasmid was transfected at 0.25 *μ*g/8^10^ cell, H: the plasmid was transfected at 0.5 *μ*g/8^10^ cell, WT: wild type, MT: mutant type. ^+^*P*<0.05 *versus* control; *P<0.05, **P<0.01 *versus* H_2_O_2_+vector; ^##^*P*<0.01, ^###^*P*<0.001 *versus* H_2_O_2_+MRTF-A. *n*=4. Data are representative of four independent experiments

To further determine the effects of HDAC5 and p300 on MRTF-A-mediated effects, we transfected HDAC5 and MRTF-A or p300 and MRTF-A together. As shown in Figures 6a–d, p300 enhanced the upregulation effect of MRTF-A on Bcl-2 and Mcl-1 mRNA and protein expression. Transfection of p300 together with MRTF-A enhanced the activity of MRTF-A on transcription of Bcl-2 and Mcl-1 via the CArG box promoter in a dose-dependent manner (Figures 6e and f). However, the transcriptional activity effect of MRTF-A on Bcl-2 and Mcl-1 was inhibited by combined transfection with HDAC5 compared with MRTF-A transfection alone (Figures 6e and f). Mutation of the CArG box abolished the effects of p300 and HDAC5.

## Discussion

Using an I/R model in rats, this study revealed that at 6 h after reperfusion, HDAC5 expression was upregulated, whereas the expression of p300 and MRTF-A as well as its target genes Bcl-2, and Mcl-1 were downregulated, resulting in the apoptosis of neurons. HDAC5 and p300 exhibited the opposite effects on both MRTF-A-mediated signaling transduction and anti-apoptotic effects on cortical neurons.

Apoptotic cerebral neurons are characterized by progressive cell death and usually appear in the peri-infarct zone after transient global ischemia, causing ischemia/reperfusion damage.^11^ Cerebral neuron apoptosis can cause hemiplegia, death, or cognitive impairment after stroke. In the present study, we confirmed that I/R could indeed induce the apoptosis of cortical neurons. It is well established that reactive oxygen species are an important inducer for cell apoptosis during the I/R period.^29^ Therefore, we used H_2_O_2_ to treat cortical neurons *in vitro* to mimic the conditions of I/R *in vivo*. The finding is in line with previous findings showing that the H_2_O_2_ could cause apoptosis of cortical neurons.

HDAC5 is abundantly expressed in the brain and has been implicated in the regulation of neurodegeneration.^30^ One recent study has suggested a possibility that intracellular translocation of HDAC5 is able to inhibit neuronal cell apoptosis induced by NMDA.^25^ HDAC5 is also known to bind to p53 and abrogate K120 acetylation, resulting in the preferential recruitment of p53 to pro-arrest and antioxidant targets during early phases of stress.^31, 32^ As a member of the HATs, p300 also modulates both histone and non-histone acetylation. In addition, p300 modifies H3 acetylation to promote gene translation,^33, 34^ acetylates PCNA to link its degradation with nucleotide excision repair synthesis,^35^ and also modifies anti-apoptotic protein p53 acetylation and inhibits the anti-apoptotic effects of p53.^36^ Based on these previous studies, it is known that HDAC5 is an anti-apoptotic protein, whereas p300 is an apoptotic protein. Interestingly, our results show that HDAC5 was upregulated, whereas p300 was downregulated during I/R, which is on contrary to some previous reports. The activity of HDAC5 and p300 were positive related with the protein expression. Therefore, both of HDAC5 and p300 In *in vitro* study, HDAC5 overexpression increased H_2_O_2_-apoptosis of cortical neuron, whereas p300 inhibited the injury effects caused by H_2_O_2_. These differences observed in this compared with some previous studies might be explained by the different animal and cell models used. Nevertheless, there may be a different signaling pathway mediating the effects of HDAC5 and p300 on the apoptosis of cortical neurons induced by I/R.

MRTF-A is a nuclear transfactor which regulates the expression of SRF-dependent target genes.^37^ The expression levels of MRTF-A are increased after stimulation with different factors, such as oxLDL or transforming growth factor-*β* (TGF-*β*), and nuclear accumulation of MRTF-A is concomitantly enhanced with the increase in MRTF-A expression.^38^ MRTF-A activity has been shown to have different roles depending on the cell type, the tissue environment, and the signaling pathways in which it is involved.^39^ In this study, MRTF-A was downregulated in the brain of rats that experienced I/R and mediated the subsequent apoptosis, in line with our previous study.^8^ Previous *in vitro* study has demonstrated that MRTF-A protein expression and the transcriptional effects on the anti-apoptotic protein Bcl-2 and Mcl-1 are accomplished via binding the CArG box in the promoter region. As a nuclear transfactor, MRTF-A must translocate in the nucleus and then promote target gene expression.^40^ Therefore, MRTF-A activity should be supposed to be impacted by itself and the chromatin structure post-translational modification. The acetylation of MRTF-A should be studied in the future. MRTF-A overexpression exerted a beneficial effect on the H_2_O_2_-induced apoptosis of cortical neurons, and HDAC5 overexpression inhibited this effect, whereas p300 overexpression promoted the anti-apoptotic effect of MRTF-A via Bcl-2 and Mcl-1 transcription. These results suggest that in the brain, after experiencing I/R injury, MRTF-A expression and its transcriptional activity may be regulated by HDAC5 and p300. Future investigations should performed to determine whether histone acetylation mediated by HDAC5 and p300 could regulate the MRTF-A activity.

In summary, the present study demonstrates for the first time that HDAC5 and p300 exert an opposite regulatory effect on MRTF-A-induced apoptosis of cortical neurons during I/R.

## Materials and methods

### Animals and the middle cerebral artery occlusion/reperfusion model

Adult male Sprague–Dawley rats (weight: 200–250 g, age: 90±4 days) were bred and held at the Experimental Animal Center of Wuhan University of Technology and Science. All rats were allowed free access to food and water before the procedure was performed under optimal conditions (12/12- h light/dark with humidity 60±5%, 22±3 °C). Focal cerebral ischemia/reperfusion (I/R) injury model was produced by transient middle cerebral artery occlusion (MCAO) for 2 h followed by reperfusion for different time periods as described in our previous report.^8^ All experimental animals were randomly allocated to the following groups: sham surgery group (*n*=16); the model group with MCAO (*n*=80) was divided into three sub-groups according to different time periods of reperfusion: reperfusion for 6 h, 12 h or 24 h. Tissues from the surrounding penumbra of cortex were isolated and snap frozen in liquid N2 for molecular biology researches or fixed by 4% paraformaldehyde for morphology researches. All animal protocols were approved by the Institutional Animal Care and Use Committee and were consistent with the Guide for the Care and Use of Laboratory Animals (updated (2011) version of the NIH guidelines).

### Intracerebroventricular injection

The adult rats, weighing 220–250 g, were anesthetized with chloral hydrate and positioned in a stereotactic apparatus (Zhenghua biological instrument equipment co., Ltd). LV-MRTF-A-siRNA or LV-negative-EGFP were injected intracerebroventricularly (i.c.v.) into the right lateral ventricle using a 10 *μ*l syringe at a rate of 1 *μ*l/min and remaining in place for 3 min after each injection. The coordinates for the stereotaxic infusion were −2.0 mm dorsal/ventral, −2.0 mm lateral, and −0.92 mm anterior/posterior from the bregma (George Paxinos 2001). Animals were allowed to recover from surgery and returned to their home cages until the time of their experimental end point.^41^

### Terminal deoxynucleotidyl transferase dUTP nick end labeling (TUNEL)

Brain sections were labeled with DeadEnd Fluorometric TUNEL System (Promega, Madison, WI, USA) or *in situ* cell death detection kit by DAB staining (Roche, Basel, Switzerland), following the manufacturer's manual as described previously.^8^ The sections were observed on a Leica DMIRE2 inverted fluorescent microscope.

### Immunostaining and binary image analysis

Fluorescence immunostaining on brain tissue sections was conducted as described previously.^42^ Briefly, rat tissue sections were incubated with blocking solution containing either rabbit anti-rat MRTF-A (1: 100, ab115319, Abcam, CA, USA) or mouse anti-rat HDAC5 (1:100, sc-11419, Santa Cruz Biotechnology, CA, USA) or rabbit anti-rat p300 (1:300, sc-585, Santa Cruz Biotechnology). Sections were washed with TBS for 30 min and then incubated with the following secondary antibodies: goat anti-mouse PE-conjugated IgG for HDAC5 (1:200, Molecular Probes, Oregon, USA), goat anti-rabbit PE-conjugated IgG for p300 and goat anti-mouse FITC conjugated IgG for MRTF-A (1:100, Molecular Probes). The nuclei were stained with DAPI (0.3 mmol/l in blocking solution) and mounted with Vectashield (Vector Laboratories, Burlingame, CA, USA). Brain sections stained with secondary antibody only were used as negative controls.

### Co-immunoprecipitation

Tissue were washed twice with phosphate-buffered saline (PBS) (pH 7.4) and lysed in NP40 buffer (50-mM Tris-Cl (pH 8.0), 150-mM NaCl, 1% NP40). The protein lysates were pre-cleared by the addition of 30 *μ*l of agarose beads for 30 min. Each immunoprecipitation (IP) reaction was initiated with 600 *μ*g of total protein and 4 *μ*g of MRTF-A or p300 antibody. The mixture was rotated overnight (4 °C) and (30 *μ*g) were added to each IP flowed by rotation for another 2 h. After centrifugation (1100 × *g* for 3 min), the supernatant was removed, and the pellet was washed four times in NP40 buffer. The complexes were eluted in SDS lysis buffer.

### Cell culture

Primary cortical neurons were isolated from 1-day-old newborn Sprague–Dawley rats as described.^43^ Briefly, cortical neurons were dissociated and cultured at a density of 2 × 10^6^ per plate in 35 mm plates. The cells were cultured in Neurobasal-Medium (with 15% horse serum, 2.5% fetal bovine serum, 100 U/ml penicillin and 100 *μ*g/ml streptomycin) and were maintained in a humidified incubator in air with 5% CO_2_. Cytosine-*β*-d-arabinofuranoside (2.5 *μ*M) was added at day 2 *in vitro* after plating to prevent the proliferation of non-neuronal cells.

### Cell transfection and H_2_O_2_ treatment

Twenty-four hours before transfection, cells were seeded at 0.3 × 10^6^ cells/cm^2^ onto 24-well plates and then transfected with various plasmids: recombination plasmid pcDNA3.1-rMRTF-A, pcDNA3.1-rHDAC6, pcDNA3.1-rp300 (Genechem biotechnology Company, Shanghai, China); the luciferase reporter vectors driven by Mcl-1 or Bcl-2 promoter sequences which were constructed using pGL-3 vectors and their mutants, and the vector pGL-3 (Promega) was used as a control. Mcl-1 Luc and Bcl-2 Luc reporter constructs contained one (Mcl-1 Luc, -527/-536: CCTTTTATGG) or two (Bcl-2 Luc: -1296: CCTTTTTAGG; 280/301: CCAAAAAAGG) CArG boxes in their promoter sequences, respectively; the additional luciferase reporter constructs of Mcl-1 or Bcl-2 promoter containing mutations to putative CArG box were generated using the QuikChange site-directed mutagenesis kit (Stratagene, La Jolla, CA, USA). The Mcl-1 luc-CArG box was mutated from CCTTTTATGG to AATTTTATAA; the Bcl-2 luc-near-CArG box was mutated from CCTTTTTAGG to CGCGGATCCG and Bcl-2 luc-far-CArG box was mutated from CCAAAAAAGG to CCAGAGCTCG.

The plasmids were diluted with Neurobasal Medium (without serum), resulting in a final volume of 100 *μ*l transfect complexes containing 0.5 *μ*g DNA and 2.5 *μ*l FuGENE HD (Invitrogen, New York, CA, USA), which were then incubated in 5% CO_2_ at 37 °C. After 48 h, cells were placed in the culture medium containing a final concentration of 400 *μ*M H_2_O_2_ for 30 min.^44^

### Apoptosis determination

Annexin V–propidium iodide (PI) staining (Beyotime Biotechnology, Jiangsu China) was analyzed using flow cytometry. Cells were collected and washed twice with PBS, followed by resuspension in 250 *μ*l of binding buffer. Five microliters of FITC–Annexin V and 10 *μ*l of PI (20 *μ*g/ml) were added to each 100-*μ*l cell suspension. The cells were incubated at room temperature for 15 min. Subsequently, 400 *μ*l of PBS was added to the cell suspensions and the samples were analyzed by flow cytometry (Becton-Dickinson, Franklin Lakes, NJ, USA).

### HDAC5 and p300 activity detection

HDAC5 and p300 were determined in whole tissue or cell lysates. Briefly, brain tissue or cells were washed using ice cold PBS, minced and homogenized in lysis buffer. Cleared lysates were analyzed for HDAC5-specific HDACT activity or p300-specific HAT activity with commercially available kits (Genmed Scientifics Inc, Arlington, MA, USA).^45, 46^

### Western blotting

The expression levels of several proteins were detected via western blot analysis.^47^ The brain tissue part was homogenized in RIPA lysis buffer (Beyontime, Jiangsu, China) and 0.1 mmol/l phenylmethylsulfonylfluoride (PMSF) (Sigma, Missouri, USA) for immunoblotting analysis. Cells were harvested and lysed in lysis buffer (50 mM Tris-HCl, pH 7.4, 150 mM NaCl, 1.5 mM MgCl2, 10% glycerol, 1% Triton X-100, 5 mM EGTA, 20 *μ*M leupeptin, 1 mM AEBSF, 1 mM NaVO3, 10 mM NaF and 1 × protein inhibitor cocktail). Proteins were separated using 10% sodium dodecyl sulfate-polyacrylamide gel electrophoresis and transferred onto a polyvinylidene fluoride (PVDF) membrane at 100 V for 1 h. Subsequently, the membrane was incubated in TBS/T buffer (20 mM Tris-HCl, pH 7.6, 150 mM NaCl, 0.1% Tween-20) with 5% non-fat milk at room temperature for 2 h. Specific primary antibodies, included rabbit anti-rat MRTF-A (diluted 1:500), mouse anti-rat HDAC5 (diluted 1:1000), rabbit anti-rat p300 (diluted 1:1000), rabbit anti-rat Bcl-2 (diluted 1:1000), rabbit anti-rat Mcl-1 and mouse anti-rat *β*-actin (diluted 1:2000), rabbit anti-rat caspase-3 (diluted 1:1000, 9664 s, Cell Signal Technology, Danvers, CA, USA); all of the antibodies were diluted in TBST buffer (50 mM Tris-HCl, with 150 mM NaCl, 0.1% Tween-20, pH 7.4) and incubated with the PVDF membrane at 4 °C overnight. Corresponding horseradish peroxidase-conjugated secondary antibodies were subsequently incubated with the PVDF membrane for 60 min at room temperature. Signal detection was performed with an enhanced chemiluminescence reagent (Amersham Biosciences, Piscataway, NJ, USA).

### Reverse-transcription PCR (RT-PCR)

Using reverse transcription-polymerase chain reaction (RT-PCR), total RNA was isolated from cells using Trizol reagent (Invitrogen). cDNA was synthesized from 12 *μ*g of total RNA in a 20 *μ*l reverse transcription (RT) system followed by PCR amplification in a 50 *μ*l PCR system performed using an RT-PCR kit (Promega). Housekeeping gene *β*-actin was used as an RNA loading control. The PCR primer sequences were as follows: Mcl-1: sense, 5′-TCATCTCCCGCTACCTGC-3′ and antisense, 5′-ACTCCACAAACCCATCCC-3′ Bcl-2: sense, 5′-GGCATCTTCTCCTTCCAG-3′ and antisense, 5′-CATCCCAGCCTCCGTTAT-3′. PCR was conducted according to the manufacturer's instructions and the PCR products were analyzed using agarose gel electrophoresis. Gels were photographed and densities of the bands were determined with a computerized image analysis system (Alpha Innotech, San Leandro, CA). The area of each band was calculated as the integrated density value (IDV). Mean values were calculated from three separate experiments. The IDV ratios of MCL-1 or BCL-2 to *β*-actin were calculated for each sample.

### Luciferase reporter assay

Luciferase assays were performed as described previously.^48^ Forty-eight hours after transfection, luciferase activity was measured using a luciferase reporter assay system (Promega) on a luminometer (Bioteck, USA). Transfection efficiencies were normalized by total protein concentrations of each luciferase assay preparation. All experiments were performed at least three times with different preparations of plasmids and primary cells, producing qualitatively similar results. Data in each experiment are presented as the mean±S.D. deviation of triplicates from a representative experiment.

### Statistical analysis

Results are expressed as the mean±S.E.M. Data were analyzed using a *t*-test for comparisons of two groups or one-way ANOVA followed by the Tukey's test for multiple comparisons. Bonferroni test was used to analyze the data of multiple groups of I/R compared with the sham control group in the *in vivo* research. Differences were considered to be statistically significant when *P*<0.05 where the critical value of *P* was two-sided. Analyses were performed using SPSS 16.0 (SPSS Inc., Chicago, IL, USA).
