# Supplementary material for: Opposite effects of HDAC5 and p300 on MRTF-A-related neuronal apoptosis during ischemia/reperfusion injury in rats
Source: Cell Death Dis. 2017 Feb 23;8(2):e2624–. doi: 10.1038/cddis.2017.16 (PMC5386465; doi:10.1038/cddis.2017.16)
Supplement: Supplementary Material [file cddis201716x1.doc]

**Supplementary materials**

**Methods**

**Measurement of infarct volume and neurological defect scoring.** Using the Longa method,[^41^](#_ENREF_41) neurological function was scored after 2 h [ischemia](http://topics.sciencedirect.com/topics/page/Ischemia) and 24 h reperfusion using five scores. Rats with no neurological defects scored 0; left forepaws of rats with flexion, adduction, and failure to extend fully scored 1; rats exhibiting circling and rotating to the left when crawling scored 2; rats exhibiting falling to the left when standing scored 3; rats that did not walk spontaneously or were in coma scored 4; and dead rats scored 5.

The ischemic hemispheres were obtained after 2 h cerebral ischemia and 24 h reperfusion to measure the infarction volume using triphenyltetrazoliumchloride (TTC) staining as described previously.[^8^](#_ENREF_8)

**Figures**


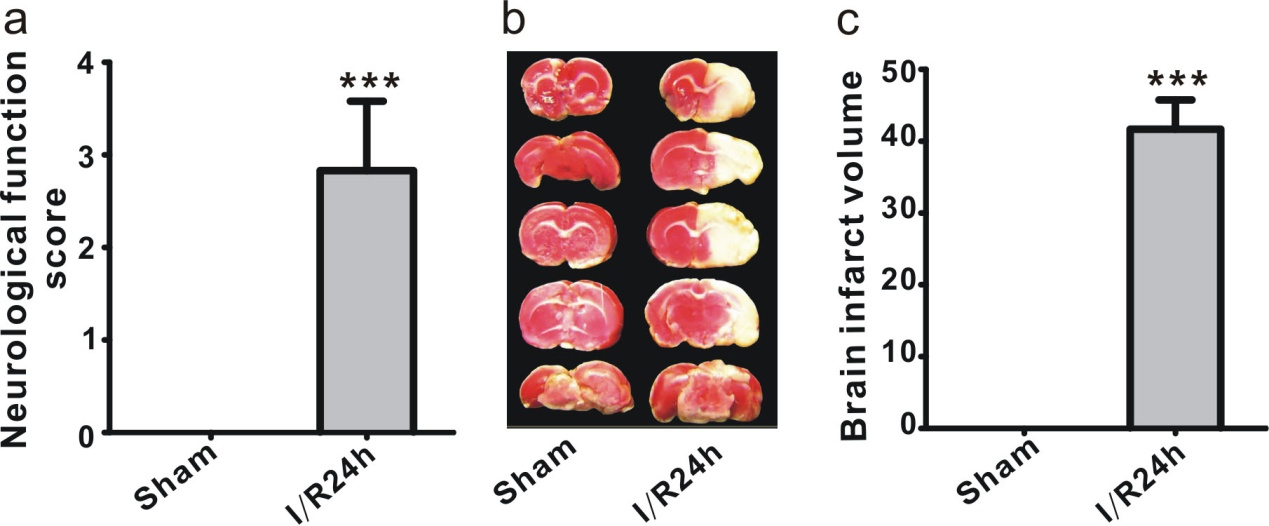


**Figure S1. The founded of MCAO rat model** The scores of Longa's behavioral test (A) and infarct volume (by TTC stained, B and C) in ischemic hemispheres after 2 h of MCAO and followed by 24 h of reperfusion in rats. Values are expressed as mean ± SEM (*n* = 6 in each group). ^***^*P <* 0*.*001 vs. sham.


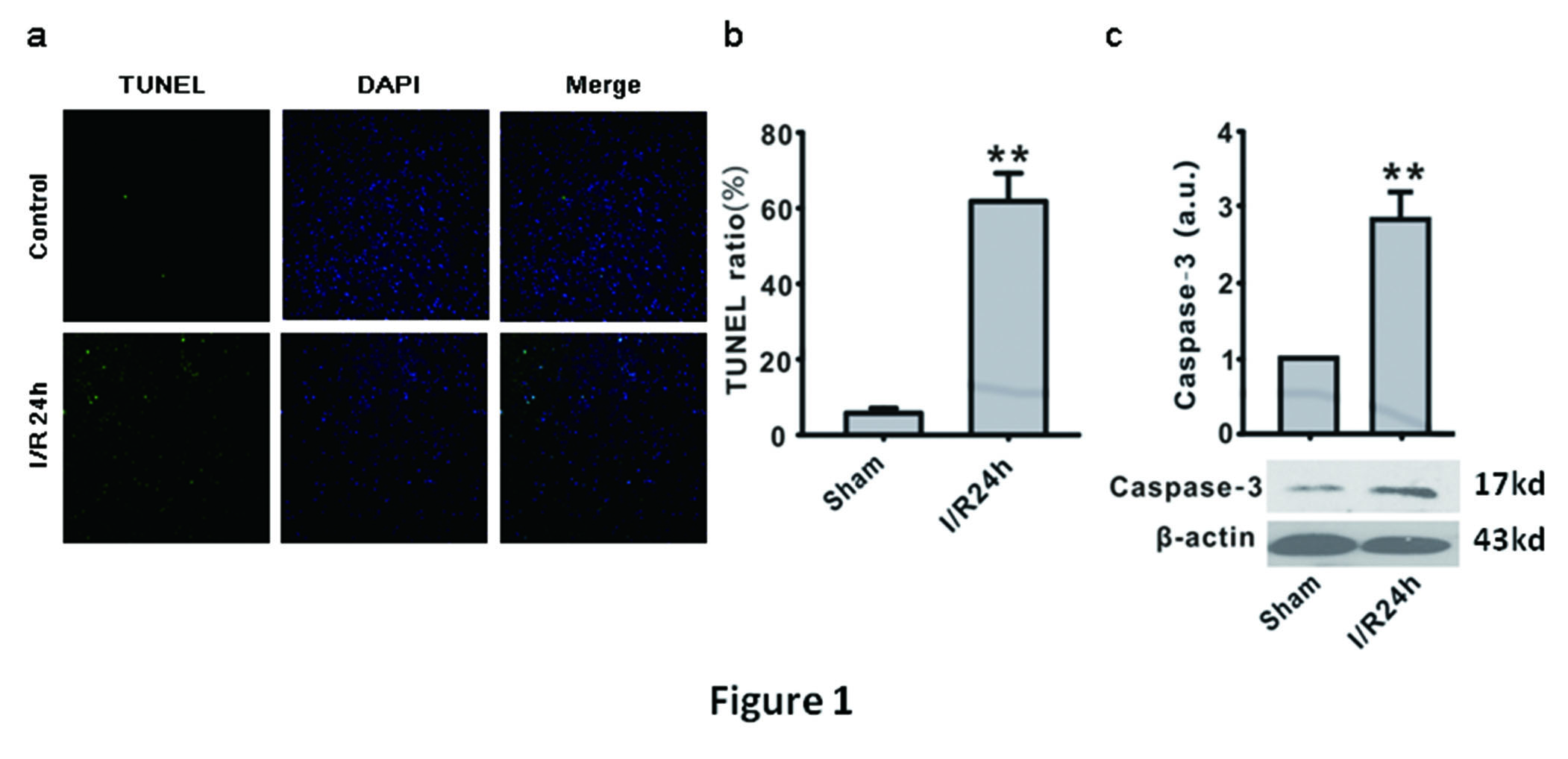
**Figure S2 Apoptosis of brain neurons induced by ischemia/reperfusion in a rat model.** Ischemia 2 h and reperfusion 24 h induced brain neuron apoptosis detected by TUNEL (a and b, 400x), and increased caspase-3 protein expression detected by western blot (c). Values are expressed as mean ± SEM (*n* = 6 in each group). ^**^*P <* 0*.*01 vs. sham.


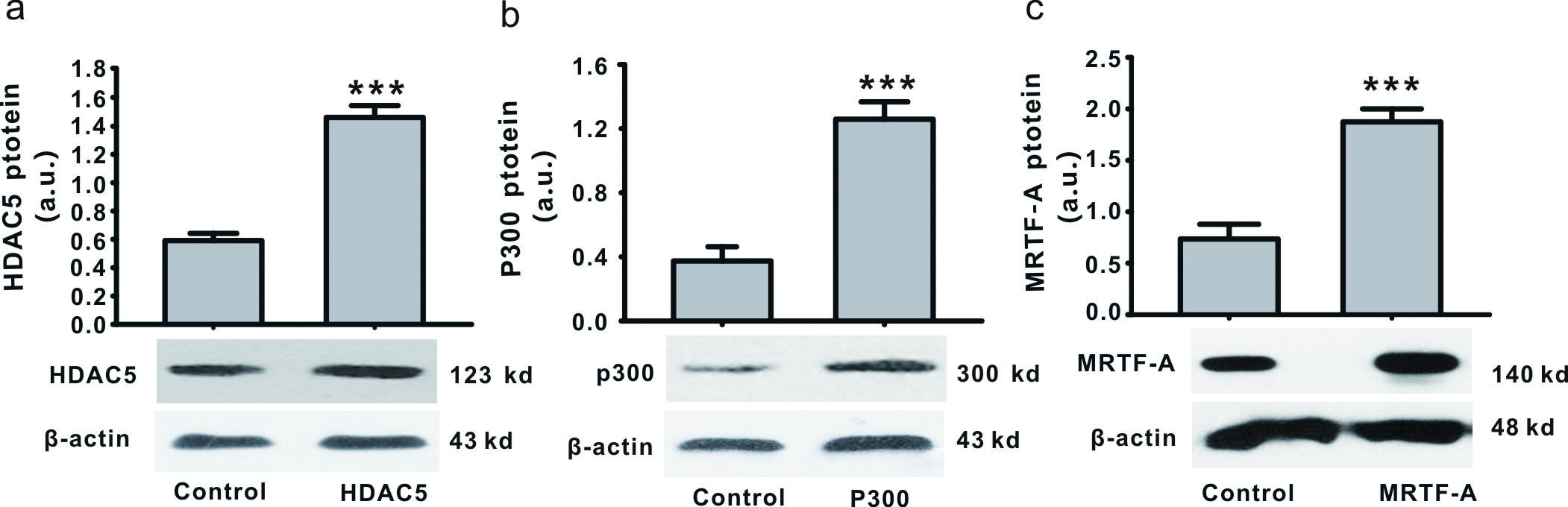


**Figure S3. HDAC5, P300 and MRTF-A overexpression cell model founded.** The cells were transfected by HDAC5 (a), P300 (b) or MRTF-A (c) recombined plasmid respectively and the protein was detected by western blot. Values are expressed as mean ± SEM (*n* = 3 in each group). ^***^*P <* 0*.*001 vs. control.
